# Factors determining anti-malarial drug use in a peri-urban population from malaria holoendemic region of western kenya

**DOI:** 10.1186/1475-2875-9-295

**Published:** 2010-10-26

**Authors:** Carren A Watsierah, Walter GZO Jura, Henry Oyugi, Benard Abong'o, Collins Ouma

**Affiliations:** 1 Great Lakes University of Kisumu, P.O. Box 2224-40100, Kisumu, Kenya; 2 Department of Zoology, Maseno University, Private Bag, Maseno, Kenya; 3 Department of Biomedical Sciences and Technology, Maseno University, Private Bag, Maseno, Kenya; 4 University of New Mexico/Centre for Global Health Research, Kenya Medical Research Institute, P.O. Box 1578-40100, Kisumu, Kenya

## Abstract

**Background:**

Interventions to reverse trends in malaria-related morbidity and mortality in Kenya focus on preventive strategies and drug efficacy. However, the pattern of use of anti-malarials in malaria-endemic populations, such as in western Kenya, is still poorly understood. It is critical to understand the patterns of anti-malarial drug use to ascertain that the currently applied new combination therapy to malaria treatment, will achieve sustained cure rates and protection against parasite resistance. Therefore, this cross-sectional study was designed to determine the patterns of use of anti-malarial drugs in households (n = 397) in peri-urban location of Manyatta-B sub-location in Kisumu in western Kenya.

**Methods:**

Household factors, associated with the pattern of anti-malarials use, were evaluated. Using clusters, questionnaire was administered to a particular household member who had the most recent malaria episode (within <2 weeks) and used an anti-malarial for cure. Mothers/caretakers provided information for children aged <13 years.

**Results:**

Stratification of the type of anti-malarial drugs taken revealed that 37.0% used sulphadoxine/pyrimethamine (SP), 32.0% artemisinin-based combined therapy (ACT), 11.1% anti-pyretics, 7.3% chloroquine (CQ), 7.1% quinine, 2.5% amodiaquine (AQ), while 3.0% used others which were perceived as anti-malarials (cough syrups and antibiotics). In a regression model, it was demonstrated that age (*P *= 0.050), household size (*P *= 0.047), household head (*P *= 0.049), household source of income (*P *= 0.015), monthly income (*P *= 0.020), duration of use (*P *= 0.029), dosage of drugs taken (*P *= 0.036), and source of drugs (*P *= 0.005) significantly influenced anti-malarial drug use. Overall, 38.8% of respondents used drugs as recommended by the Ministry of Health.

**Conclusion:**

This study demonstrates that consumers require access to correct and comprehensible information associated with use of drugs, including self-prescription. There is potential need by the Kenyan government to improve malaria care and decrease malaria-related morbidity and mortality by increasing drug affordability, ensuring that the recommended anti-malarial drugs are easily available in all government approved drug outlets and educates the local shopkeepers on the symptoms and appropriate treatment of malaria. Following a switch to ACT in national drug policy, education on awareness and behaviour change is recommended, since the efficacy of ACT alone is not sufficient to reduce morbidity and mortality due to malaria.

## Background

There has been some level of improper use of anti-malarial drugs despite the fact that *Plasmodium falciparum *malaria still remains the primary cause of morbidity and mortality amongst millions of people living in Africa [1]. The lack of proper drug use has been promoted largely over the years by the self-administration of the most common anti-malarial drugs, particularly chloroquine (CQ) [1]. In many situations, practices that could best be described as misuse of drugs have become routine, and in some cases, promoted institutionally [1]. This has further culminated into little interventions to improve the use of anti-malarial drugs. Previously, both CQ and sulphadoxine/pyrimethamine (SP) were perceived to be inexpensive, making it more cost-effective to treat presumptive conditions of malaria rather than to attempt to get microscopic confirmation, especially when this conventional method is unavailable, as commonly experienced in most parts of Africa [1].

In particular, the pattern of anti-malarial drug use in endemic areas of Africa is quite different from the pattern of use of other drugs due to the high intensity of malaria transmission in these areas. In areas of intense malaria transmission, anti-malarial drugs are given repeatedly to treat frequent fevers (even in the absence of malaria) [2]. The practice of self-medication is also increasingly becoming a major health concern. Research finding shows that many illnesses including malaria, are treated without consultations from health professionals [3]. In areas where malaria transmission is endemic, about 50-80% of people first visit private drug outlets for malaria treatment and use these anti-malarial drugs even without prescription, a practice which has resulted in patterns such as over-use, under-use and irregular use of these drugs [4].

Mortality in approximately 26,000 children and about a third of out-patient attendance in health facilities throughout Kenya annually is attributed to *P. falciparum *malaria. Adult disability due to malaria in this region translates to an economic loss of about 160 million working days per year [5]. The Kenyan National Drug Policy was established in 1994 to check on medication, prescription and promotion of rational drug use. However, the policy has never been meaningfully implemented [5]. It is, therefore, critical to effectively evaluate information on the drug use among the different population. Further dissemination of these facts to both the health authorities and the scientific communities will facilitate effective understanding and help to address the drug use patterns. The current study site, a peri-urban location within Kisumu, is situated in a *P. falciparum *holoendemic transmission area of western Kenya. Malaria in this region accounts for 40% of out-patient visits, and 40% of hospital in-patient admissions [6]. Malaria transmission occurs all year round, peaking in the rainy season months of April and May [7]. Most malaria cases in this region are managed at household level. The patterns of use of anti-malarials include self-prescription, sharing of drugs with friends and relatives and premature discontinuation of treatment course [4]. Anti-malarials are usually bought from commercial pharmacists, retail shops (e.g. grocery, general stores, and community pharmacies) or from licensed and unlicensed drug sellers. Most patients reporting at the health facilities would have at least gone through home-based treatment or local drug shops initially. Thus, seeking health care from practitioners may be due mainly to resistant, severe, or recurrent episodes [4]. Hence, there is concern that household and communities' patterns of use of anti-malarial drugs are often inadequate and inappropriate depending on the practices adopted for use of medicines not only for malaria [8]. The situation may be worse among households living in malaria endemic regions such as western Kenya, since high endemicity may mean that the population invents mechanisms for surviving constant malarial episodes rather than seek adequate healthcare. It is therefore imperative to understand how anti-malarial drugs are used in such areas. In addition, since few documented studies have been carried out to link household factors such as economic level coupled with high endemicity, with the patterns of use of anti-malarials, it is upon this background that we report on the factors determining the patterns of anti-malarial drugs use among the residents of a peri-urban region of Kisumu in Western Kenya.

## Methods

### Study setting

The current study was carried out in Manyatta-B sub-location in Winam division of Kisumu. The area consists of three villages, Lower Kanyakwar, Upper Kanyakwar and Kuoyo, which are further divided into nine smaller units depending on the sub-clan boundaries. Within the sub-clan boundaries are informal dwellings within and outside some of the homesteads. According to the last population census, the total number of inhabitants in this sub-location is 21,027 in 6,035 households [9]. The total area is 3.3 km^2 ^with a density of 6, 372 people/km^2 ^[9]. Housings consist of mostly rented rooms within an area of 10-20 m^2 ^for 4-8 persons in a household. These individuals live in a congested and miserable conditions, with considerable lack of the basic necessities of life, such as clean piped-water and proper housing conditions [10]. The density of the population and the closely-packed housing conditions contributes to poor drainage; hence, stagnant water, which form good breeding grounds all year round for malaria vectors [11].

### Study participants

In order to determine the patterns of use of anti-malarial drugs in the study population at a point in time, residents living within households (n = 397) in the above described peri-urban region of Kisumu in western Kenya, were recruited. This was a descriptive cross-sectional survey in which sampling was carried out using cluster method as previously described [12]. A total of 6,035 households in nine clusters were sampled in order to achieve the required sample size. Data was collected through interviews, using structured questions in selected households in which diagnosed or self-reported episodes of malaria occurred within the last two weeks prior to the interview dates.

Included in the study were individuals living in the above described households and who had experienced malaria and used anti-malarial or other drugs perceived by them as anti-malarial, due to self/presumptive or laboratory diagnosis within the previous two weeks. In the absence of laboratory diagnosis, malaria according to residents in the study area was defined by either the presence or mixed symptoms of fever, diarrhoea, vomiting and pain in the joints. However, if more than one individual in a household had experienced malaria and had used anti-malarial or other drugs viewed or perceived by them as an anti-malarial within the described period (i.e. two weeks), then focus was on the most current episode, while the rest were excluded. This strategy was adopted so as to capture the most recent pattern of anti-malarial drug use and to minimize errors associated with recall. If no one had experienced malaria episodes within the previous two weeks, the interviewer moved to the next household in the cluster. Correct drug use was assessed by questionnaires through identification of the drug used and whether or not the respondents used it as per the prescription. The study was approved by the ethical and scientific review committees at the Kenya Medical Research Institute and the Great Lakes University of Kisumu Ethical Review Board (GERB), Kenya.

### Sampling design

This study employed cluster sampling method in selecting the households in the study area. A total of nine clan regions were chosen as clusters. The study units comprised households within the cluster that had its member experiencing malaria in the last two weeks. The probability of selection of the primary sampling units for each cluster was proportional to the estimated sample size. From each cluster, households were then selected on continuous basis until the total number (n = 397) required was achieved.

### Research procedure

The quantitative data, which was collected from 397 households was performed through interviews using structured questions. The tool of data collection was a pre-prepared questionnaire, which was administered to the particular household member, who had previous malaria episode and used anti-malarial or drug perceived to be an anti-malarial. However, in cases of children below the age of 13 years, mothers or caretakers provided the information. Using clusters, households with malaria episodes were established first through identification interview, which involved all the households starting with exactly the first household visited in the cluster. The interview was then repeated in all the households identified until the required number was achieved. As stated above, particular emphasis was on the most recent episode of self/presumptive or laboratory diagnosed malaria in the household. Research/field assistants (n = 5) who were involved in data collection, used drug charts containing samples of commonly used anti-malarial drugs to aid in recall and to validate reports. In addition, remaining packages or tablets were reviewed where possible.

Prior to the actual study, the field assistants' who were themselves residents of the study sub-location and with a minimum of form four level of education, were interviewed and recruited as enumerators. They were required to be fluent in English, Swahili and DhoLuo languages. Training of the research assistants on survey interviewing techniques was carried out for one day followed by a second day of pre-testing of survey tools and methodologies in a neighbouring community with the same characteristics. Based on the experiences and results of the pre-test, further re-training and refining of techniques of interviewing and modification of research tool was performed.

### Statistical analyses

Statistical analyses were performed using SPSS (Version 15.0). Logistic regression analysis between the independent and dependant variables was used to identify variables associated with pattern of use of anti-malarial drugs. The variables included in the logistic regression model included; demographic (age, gender, marital status, household head, and household size); socio-economic (household breadwinner, source of income, household monthly income, education level and ability to read) and environmental (source of drugs, distance to source of drugs, and availability of anti-malarial drug at source) factors. The variables that were significantly associated with pattern of use (drugs taken, duration, dosage and source of drugs) were further analyzed using Chi-square tests. Proportions on knowledge, drug-taking behaviour and correctness of patterns of use were determined. A *P *≤ 0.05 was considered statistically significant.

## Results

### Demographic characteristics of the study participants

A total of 397 households were included in the analyses to determine factors associated with anti-malarial drugs use patterns. Overall, males were 183 (46.1%) while females were 214 (53.9%) (Table 1). In terms of age, those who were aged <13 years were 262 (66.0%) while 135 (34.0%) were ≥13 years. The distribution of individuals per household were as follows; 1-2 individuals [81 (20.4%)], 3-5 individuals [215 (52.4%)], and ≥6 individuals [101 (25.4%)]. Households headed by husbands were 267 (67.3%); wives 76 (19.1%) and caretakers 51 (12.8%), while 3 (0.8%) were headed by others. Respondents who were married were 88 (22.8%); single 23 (5.8%) and widowed 12 (3.0%) while underage individuals (i.e. below 15 years and not marriageable) were 271 (68.4%).

**Table 1:** Demographic characteristics and the use of three major anti-malarial drugs

Demographic factors	*Drugs taken*				*Duration of use*		*Dosage of use*	
	
		*SP*	*ACT*	*Quinine*	*Correct*	*Incorrect*	*Correct*	*Incorrect*
Age	<*13 years*	98 (37.4%)	86 (32.8%)	20 (7.6%)	159 (60.7%)	103 (39.3%)	202 (77.1%)	60 (22.9%)
	≥*13 years*	49 (36.3%)	41 (30.4%)	8 (5.9%)	69 (51.1%)	51 (48.9%)	102 (75.6%)	33 (24.4%)
Gender	*Male*	67 (36.6%)	60 (32.8)	16 (8.7%)	104 (56.8%)	79 (43.2%)	146 (79.8%)	37 (20.2%)
	*Female*	80 (37.4%)	67 (31.3%)	12 (5.6%)	124 (57.9%)	90 (42.1%)	158 (73.8%)	56 (26.2%)
Marital status	*Single*	7 (30.4%)	6 (26.1%)	2 (8.7%)	14 (60.9%)	9 (39.1%)	18 (78.3%)	5 (21.7%)
	*Married*	31 (31.2%)	31 (35.2%)	3 (3.4%)	50 (56.8%)	38 (43.2%)	67 (76.1%)	21 (23.9%)
	*Separated*	1 (50.0%)	0	0	0 (0.00%)	2 (100%)	1 (50.0%)	1 (50.0%)
	*Widowed*	5 (41.7%)	0	3 (25.0%)	8 (66.7%)	4 (33.3%)	8 (66.7%)	4 (33.3%)
	*Underage*	102 (37.6%)	90 (33.2%)	20 (7.4%)	155 (57.3%)	116 (42.8%)	209 (77.1%)	62 (22.9%)
HH head	*Husband*	108 (40.4%)	88 (33.0%)	13(4.9%)	161 (60.3%)	106 (39.7%)	205 (76.8%)	62 (23.2%)
	*Wife*	24 (31.6%)	25 (32.9%)	9 (11.8%)	35 (46.1%)	41 (53.9%)	58 (76.3%)	18 (23.7%)
	*Caretaker*	14 (27.5%)	13 (25.5%)	6 (11.8%)	30 (58.8%)	21 (41.2%)	39 (76.5%)	12 (23.5%)
	*Others*	1 (33.3%)	1 (33.3%)	0	1 (33.3%)	2 (66.7%)	2 (66.7%)	1 (33.3%)
HH size	*1-2*	32 (39.5%)	27 (33.3%)	4 (4.9%)	42 (51.9%)	39 (48.1%)	59 (72.8%)	22 (27.2%)
	*3-5*	83 (38.6%)	65 (30.2%)	16 (7.4%)	127 (59.1%)	88 (40.9%)	167 (77.7%)	48 (22.3%)
	*6 and above*	32 (31.7%)	35 (34.7%)	8 (7.9%)	59 (58.4%)	42 (41.6%)	78 (77.2%)	23 (22.8%)

HH- Household, SP- Sulphadoxine pyremethamine, ACT- Artemether-based combined therapies. Proportionality determined by Chi-square test. From the Table, most respondents used SP and ACT relative to other drugs, with close variations in their use. In addition, more than half of the respondents took the drugs within the correct duration in each case considered, except for households who were headed by wives (46.1%). Furthermore, majority in the different categories used the correct dose as opposed to lower proportions of individuals who used the incorrect dose.

Levels of education varied in that approximately 13.4% had achieved primary education, 12.1% secondary education, 2.5% had post-secondary education, while 14.1% were still in school. In addition, 3.0% did not have any form of education while 54.9% were underage children (i.e. <6 years and not started any formal education). The primary breadwinners for most households were husbands (69.9%), wives (15.9%) and others (14.2%). Self-employment (37.9%) was the main source of income while petty business (25.5%), salary (20.2%), casual work (15.4%), and farming and the other sources of income were (1%). The respondents with monthly income of below KShs. 4,500 were 41.2%, between KShs. 4,500-9,000 were 46.5%, while over KShs. 9,000 were 12.4%. A total of 79.8% and 20.2% of respondents were able and unable to read prescriptions, respectively (Table 1).

In terms of drug use, further findings revealed that most respondents used SP and ACT relative to other drugs, with close variations in their use. In addition, more than half of the respondents took the drugs within the correct duration in each case considered, except for households who were headed by wives (46.1%). Furthermore, majority in the different categories used the correct dose as opposed to lower proportions of individuals who used the incorrect dose (Table 1).

### Sources of anti-malarials in the peri-urban population

In order to determine whether the drugs were acquired from the correct sources with the right prescriptions, respondents were asked about the sources of their drugs. Results indicated that a total of 30.5% of drugs were from government institutions, 30.3% from nearby shops, 18.3% from pharmacies/chemists, 16.4% from private clinics, 1.0% from street vendors, 1.5% from friends/neighbours/relatives, and 2.0% were left-over drugs in the households.

Since the source can determine the type of drug used, the study further assessed the pattern of individual drug used from the above sources. For quinine, the main source was government health institutions (66.4%) and private clinics (33.6%). The ACT drugs were mainly sourced from government health institutions (60.0%), pharmacy/chemists (25.2%) and private clinics (14.8%). The main source of SP drugs was shops/kiosks (70%) while pharmacy/chemists (21.2%) and private clinics (8.8%). Other sources of anti-malarials included street vendors, friends/relatives/neighbours and left-over medicine in the households whose proportions were <20% for each of the categories.

Distance to the source of an anti-malarial drug was measured by the time taken to reach the source while walking. Respondents who took less than 30 minutes were 57.4% while 42.6% took more than 30 minutes to reach the source of drug. Furthermore, 48.0% of respondents accessed the anti-malarial drugs at the respective places/sources because those were the types of drugs that were available and the source was a shorter distance to the respondent (Table 2).

**Table 2:** Socio-economic factors and the use of three major anti-malarial drugs

Socio-economic factors	*Drugs taken*				*Duration of use*		*Dosage of use*	
	
		*SP*	*ACT*	*Quinine*	*Correct*	*Incorrect*	*Correct*	*Incorrect*
Education level	*Primary*	19 (35.8%)	18 (34.0%)	5 (9.4%)	28 (52.8%)	25 (47.2%)	44 (83.0%)	9 (17.0%)
	*Secondary*	19 (39.6%)	15 (31.3%)	1 (2.1%)	5 (50.0%)	21 (43.8%)	36 (75.0%)	12 (25.0%)
	*Post-Secondary*	5 (50.0%)	3 (30.0%)	0	27 (56.3%)	5 (50.0%)	6 (60%)	4 (40.0%)
	*Still in school*	17 (30.4%)	20 (35.7%)	4 (7.1%)	37 (66.1%)	19 (33.9%)	45 (80.4%)	11(19.6%)
	*None*	4 (33.3%)	0	1 (8.3%)	10 (83.3%)	2 (16.7%)	8 (66.7%)	4(33.3%)
	*Under 6*	83 (38.1%)	71 (32.6%)	17(7.8%)	121 (55.5%)	97 (44.5%)	165 (75.7%)	53(24.3%)
HH breadwinner	*Husband*	105 (37.9%)	97 (35.0%)	16 (5.8%)	164 (59.2%)	113 (40.8%)	219 (79.1%)	58 (20.9%)
	*Wife*	21 (33.3%)	18 (28.6%)	4 (6.3%)	30 (47.6%)	33 (52.4%)	45 (71.4%)	18 (28.6%)
	*Others*	21 (37.3%)	11 (20.4%)	8 (14.8%)	34 (61.1%)	22 (38.9%)	39 (68.5%)	17 (31.5%)
HH inc. source	*Salaried*	27 (33.8%)	29 (36.3%)	6 (7.5%)	50 (62.5%)	30 (37.5%)	65 (81.3%)	15 (18.8%)
	*Self employed*	53 (35.3%)	47 (31.3%)	7 (4.7%)	96 (64.0%)	54 (36.0%)	110 (73.3%)	40 (26.7%)
	*Petty business*	42 (41.6%)	27(26.7%)	11 (10.9%)	51 (50.5%)	50 (49.5%)	79 (78.2%)	22 (21.8%)
	*Casual worker*	24 (39.3%)	23 (37.7%)	4 (6.6%)	29 (47.5%)	32 (52.5%)	47 (77.0%)	14 (23.0%)
	*Farming*	1 (50.0%)	1 (50.0%)	0	1 (50.0%)	1 (50.0%)	1(50.0%)	1 (50.0%)
	*Others*	0	0	0	1(50.0%)	1 (50.0%)	1 (50.0%)	1 (50.0%)
HH monthly inc.	*Below Ksh.4500*	56 (34.4%)	48 (29.4%)	11 (6.7%)	85 (52.1%)	78 (47.9%)	115 (70.6%)	48 (29.4%)
	*Ksh.4500-9000*	74 (40.2%)	58 (31.5%)	12 (6.5%)	111 (60.3%)	73(39.7%)	147 (79.9%)	37 (20.1%)
	*Over Ksh. 9000*	17 (35.4%)	21 (43.8%)	4(8.3%)	30 (62.5%)	18 (37.5%)	40 (83.3%)	8 (16.7%)
Ability to read	*Able to read*	121 (38.2%)	114 (36.0%)	20 (6.3%)	181 (57.1%)	136 (42.9%)	246 (77.6%)	71 (22.4%)
	*Unable to read*	26 (32.9%)	13 (16.5%)	7 (8.9%)	46 (58.2%)	33 (41.8%)	57 (72.2%)	22 (27.8%)

Data are presented as proportions (n,%). HH-Household, SP- Sulfadoxine pyremethamine, ACT- Artemether-based combined therapies, inc.-Income. From the results above the drugs that were taken by majority of the respondents were SP and ACT in all the socio-economic factors considered, with very small differences in proportions in each category. In addition, averagely half of the households had used drugs for correct duration, unlike households whose breadwinners were wives (47.6%) and those who depended on casual work as the main source of income (47.5%). Moreover, most households took correct dosages of drugs with majority scoring more than 70% in each socio-economic factor considered.

### Association between demographic, socio-economic, and environmental factors and anti-malarial drug use patterns

In order to determine factors influencing patterns of use of anti-malarial drugs in the peri-urban population, a logistic regression analysis was performed. The independent variables included demographic (age, gender, marital status, household head, and household size), socio-economic (household breadwinner, source of income, household monthly income, education level and ability to read) and environmental factors (source of drugs, distance to source of drugs, and availability at source). The above factors were regressed against the outcome variables (i.e. drugs taken, duration of use and dosage/frequency of use). Age was a significant factor in determining duration of anti-malarial use since many respondents (60.7%) who were <13 years took drugs for correct duration as opposed to respondents who were ≥13 years (39.3%) (β = 0.179, *P *= 0.050). The household head determined duration of use since 60.3% of households headed by husbands took anti-malarial drugs for the correct duration as compared to other household headed by either wives or any other person other than husbands (β = 0.093, *P *= 0.047%). In addition, household size significantly influenced the types of anti-malarial drugs used (β = 0.092, *P *= 0.049) (Table 3). A higher proportion of the respondents whose household sizes were 3-5 (68.9%) or ≥6 (20.1%) used SP as compared to households with 1-2 individuals (11.0%) (*P *= 0.015). Furthermore, household source of income significantly influenced duration for use (β = 0.097, *P *= 0.050) since a higher proportion of respondents who were salaried (21.9%) or self-employed (38.6%) used drugs within the specified duration relative to those with other sources of income (*P *= 0.017). In addition, household monthly income significantly influenced dosage of the drugs used (β = 0.113, *P *= 0.034) (Table 3), primarily due to the fact that higher proportions of respondents with an income salary of KShs. 4500-9000 (48.5%) took the correct dosage of drugs as opposed to individuals with other incomes (*P *= 0.020).

**Table 3:** Demographic, socio-economic and environmental factors associated with patterns of use of anti-malarial drugs

	Dependent variables		
	
	Drugs taken	Dosage of use	Duration of use
**Independent variables**			

Age (A)	β = 0.113, *P *= 0.230	β = 0.006, *P *= 0.949	β = 0.179, ***P *= 0.050**
Gender (G)	β = 0.008, *P *= 0.872	β = 0.061, *P *= 0.235	β = 0.018, *P *= 0.722
Marital status (MS)	β = 0.101, *P *= 0.341	β = 0.115, *P *= 0.279	β = 0.105, *P *= 0.322
Household size (HHS)	β = 0.092, ***P *= 0.049**	β = 0.027, *P *= 0.606	β = 0.008, *P *= 0.873
Household head (HHH)	β = 0.019, *P *= 0.726	β = 0.011, *P *= 0.841	β = 0.093, ***P *= 0.047**
Education level (EL)	β = 0.018, *P *= 0.828	β = 0.132, *P *= 0.104	β = 0.057, *P *= 0.486
Household breadwinner (HHB)	β = 0.008, *P *= 0.883	β = 0.086, *P *= 0.113	β = 0.071, *P *= 0.188
Household income source (HIS)	β = 0.028, *P *= 0.597	β = 0.010, *P *= 0.846	β = 0.097, ***P *= 0.050**
Household monthly income (HHI)	β = 0.037, *P *= 0.486	β = 0.113, ***P *= 0.034**	β = 0.064, *P *= 0.233
Ability to read (AR)	β = 0.059, *P *= 0.268	β = 0.005, *P *= 0.926	β = 0.030, *P *= 0.570
Source of drug (SD)	β = 0.113, ***P *= 0.036**	β = 0.013,***P *= 0.005**	β = 0.029, *P *= 0.596
Distance to source (DS)	β = 0.049, *P *= 0.373	β = 0.021, *P *= 698	β = 0.058, *P *= 0.293
Availability at source (AS)	β = 0.092, *P *= 0.046	β = 0.002, *P *= 965	β = 0.060, *P *= 0.252

Logistic regression analysis between the independent and dependant variables was used to identify variables associated with pattern of use of anti-malarial drugs, including demographic, socio-economic and environmental factors. *P*-values in bold were statistically significant at *P *≤ 0.05. b = standard co-efficient. The result above shows that age, household size, source of drugs and availability of drugs at the source work together to influence types of anti-malarial that are taken in the households. Furthermore, household monthly income and source of drug work together to influence dosage of use; while age, household head and household income source work together to influence duration of use of these drugs.

Additional analyses revealed that the source of drugs significantly predicted pattern of anti-malarial drug use by determining dosage (β = 0.013, *P *= 0.005) and drug taken (β = 0.113, *P *= 0.036). This observation is in line with the fact that higher proportions of respondents who took the correct dosage of drug either acquired their drugs at government institutions (29.3%) or pharmacy/chemists (24%) relative to other sources (*P *= 0.004). Moreover, availability at source significantly influenced anti-malarial drug taken (β = 0.092, *P *= 0.046) (Table 3). Taken together, these results demonstrate that in this peri-urban population of Kisumu, age, household head, household size, source of income, level of monthly income, the source of anti-malarial and availability at the source significantly influence pattern of anti-malarial use.

## Discussion

The importance of understanding how anti-malarial drugs are used in a community and consequently how their use might be improved draws a considerable interest and concern in translating anti-malarial efficacy into effectiveness in various settings in malaria-affected communities. This study was designed to investigate the demographic, socio-economic and environmental factors that influence anti-malarial drug use in a peri-urban region within Kisumu in western Kenya. The findings presented here demonstrate that age, household head, household size, source of income, level of monthly income, the source of anti-malarial and availability at the source significantly influence pattern of anti-malarial use.

Age influenced pattern of anti-malarial use by influencing duration for use. A total of 60.7% individuals <13 years used drugs for correct duration while only 51.1% ≥13 years of age used drugs for correct duration. Higher proportion of individuals (i.e. children) <13 years taking drugs for correct duration may be attributed to the fact that they are dependent on caretakers in making health decisions and supervisions. Further analyses demonstrated that gender and marital status did not influence the pattern of anti-malarial use. This finding is consistent with a previous study carried out in Turkey to determine knowledge on malaria and behaviour in endemic rural area in which there were no relationships between adherence and race, gender and marital status [13]. However, the current findings and the previous ones [13] are inconsistent with a study carried out in Sudan among college students on self-medication practices with antibiotics and anti-malarial drugs [14] and in a community-based cross-sectional survey in Tulu District in southern-central Ethiopia [15], in which it was demonstrated that treatment-seeking behavior for malaria was not affected by age.

Household head influenced the pattern of use of anti-malarial drugs by influencing the duration for use. This observation could be attributed to the fact that husbands, being majority of the household heads were also the primary breadwinners, and hence, were influencing health decisions in the household. In addition, the household size influenced patterns of use of anti-malarial drugs since it determined the anti-malarial drugs taken in the household. Households with 1-2 individuals had a tendency to take the correct dosage within the correct duration relative to households with three or more individuals, which demonstrated an irregular pattern. This could be attributed to the fact that larger household sizes, due to economic strain, need more finances to maintain, hence would prefer cheaper anti-malarials, which then affects the type, dosage and duration of use.

Assessment of socio-economic factors that could influence anti-malarial drug use revealed that households with a monthly income of KShs. 9000 took correct dosages as opposed to those with other incomes. Furthermore, most households which depended on salary and on self-employment took drugs for correct duration as opposed to households with other sources of income. These results are comparable to a study in Uganda [16] and Ethiopia [17], which were designed to determine healthcare-seeking behaviour for malaria treatment in which higher income households frequently obtained their drugs from commercial drug stores because they could afford them. In line with this argument, higher income households in the current study were also able to afford the drugs and hence use correct doses.

Assessment of environmental factors that can influence anti-malarial drug use revealed that the source of drugs and availability at source influenced pattern of use by influencing dosage and drugs taken, respectively. This observation was mainly because higher proportions of the respondents who took the correct dosage of drug either acquired the drugs from government health facilities or from pharmacy/chemists as opposed to other sources. In addition, availability of drugs determined which drugs are obtainable for use. These findings are consistent with findings in a study carried out in Southern Ethiopia on self-treatment of malaria in rural communities, where it was demonstrated that anti-malarial drugs were bought from markets or any shops because it was close to home (43.6%), the drugs were available (25.8%), the shops were easily accessible (20.9%), and waiting time was short (11.5%) [17]. In a different study in Kenya, it has been demonstrated that utilization of health facilities decreased slightly with increasing distance of participants' place of residence to health facilities for up to 6 km [18]. In line with these previous observations, findings in this study indicate that large segment of the households in the peri-urban population are living within a short radius from health facilities, or alternative sources of anti-malarial drugs. Consequently, it is reasonable to suggest that this may produce a levelling effect and may not be an influencing factor in pattern of use of anti-malarial. In terms of source of drugs, most respondents in the current study obtained their anti-malarial drugs from government health facilities, contrary to previous studies in other similar *P. falciparum *endemic regions in which anti-malarial drugs were obtained from local shops [19,20]. However, the findings from the current study concurs with the behavioural patterns observed in a study in Kipsamoite area, which is a highland region of Kenya (with unstable malaria transmission pattern) in which medical facilities were the most common initial treatment source for both adults (66.0%) and children (66.7%). Local shops were the most popular alternative, providing initial treatment for 19.0% of adults and 30.3% of children [21]. The observed patterns in the current study could be due to improved availability and provision of subsidized anti-malarial drugs at government facilities. It is important to note that even though local shops were not the most popular source of anti-malaria drugs in the current setting, they were the most frequently sought source after government medical facilities. As a result, a significant portion of the population received anti-malarial drugs for perceived malaria from the local shops. These findings indicate that, despite increased reliance on government facilities in this peri-urban area, local shops are an important source of anti-malarial drugs and should be considered when designing treatment and intervention programmes.

## Conclusion

In conclusion, there is potential need by the Kenyan government to improve malaria care and decrease the malaria-related morbidity and mortality by increasing drug affordability, ensuring that the recommended anti-malarial drugs are easily available in all government approved drug outlets and in addition, educate the local shopkeepers on the symptoms and appropriate treatment of malaria. Additional efforts should be made in further organizing awareness campaigns to educate the locals on the clinical symptoms of malaria. Such educational interventions have been shown to change malaria treatment-seeking behaviour in other malaria endemic areas [22-24].

## Competing interests

The authors declare that they have no competing interests.

## Authors' contributions

CAW designed, carried out the survey studies in the peri-urban population and participated in the drafting of the manuscript. WGZOJ and CO designed the study and participated in the drafting of the manuscript. HO and BA performed the statistical analysis. All authors read and approved the final manuscript.
